# Creating Images With the Stroke of a Hand: Depiction of Size and Shape in Sign Language

**DOI:** 10.3389/fpsyg.2018.01276

**Published:** 2018-07-31

**Authors:** Jenny C. Lu, Susan Goldin-Meadow

**Affiliations:** ^1^Department of Psychology, The University of Chicago, Chicago, IL, United States; ^2^Departments of Psychology and Comparative Human Development, The University of Chicago, Chicago, IL, United States

**Keywords:** depiction, depicting constructions, iconic mouth movements, gesture, iconicity

## Abstract

In everyday communication, not only do speakers describe, but they also depict. When depicting, speakers take on the role of other people and quote their speech or imitate their actions. In previous work, we developed a paradigm to elicit depictions in speakers. Here we apply this paradigm to signers to explore depiction in the manual modality, with a focus on depiction of the size and shape of objects. We asked signers to describe two objects that could easily be characterized using lexical signs (Descriptive Elicitation), and objects that were more difficult to distinguish using lexical signs, thus encouraging the signers to depict (Depictive Elicitation). We found that signers used two types of depicting constructions (DCs), conventional DCs and embellished DCs. Both conventional and embellished DCs make use of categorical handshapes to identify objects. But embellished DCs also capture imagistic aspects of the objects, either by adding a tracing movement to gradiently depict the contours of the object, or by adding a second handshape to depict the configuration of the object. Embellished DCs were more frequent in the Depictive Elicitation context than in the Descriptive Elicitation context; lexical signs showed the reverse pattern; and conventional DCs were equally like in the two contexts. In addition, signers produced iconic mouth movements, which are temporally and semantically integrated with the signs they accompany and depict the size and shape of objects, more often with embellished DCs than with either lexical signs or conventional DCs. Embellished DCs share a number of properties with embedded depictions, constructed action, and constructed dialog in signed and spoken languages. We discuss linguistic constraints on these gradient depictions, focusing on how handshape constrains the type of depictions that can be formed, and the function of depiction in everyday discourse.

## Introduction

In everyday communication, not only do people use words to convey their thoughts and actions, but they also often iconically demonstrate what they are thinking or seeing. For example, consider two accounts of a bicycle accident:

(1) The bike crashed into the woman.
(2) The bike hit the woman, *BAAAM* [iconic gesture in which one hand collides with the other].

In (1) the speaker describes the event using only conventional lexical items, and conveys the fact that the collision was violent with the word “crash.” In (2) the speaker describes the event with a less evocative lexical item (“hit”) but adds information about the severity of the collision with vocal onomatopoeia and the vowel elongated, *BAAM*, accompanied by an imagistic gesture depicting the crash. Instead of simply describing the event as in (1), the speaker in (2) combines two modes of representations—conventional signs and spontaneous depictions. The second utterance may do a better job of evoking a sensory image of the event, allowing one to imagine just how bad the collision was.

Speakers often combine different communicative devices—words, gestures, enactments—to produce multi-modal “composite utterances” (Enfield, 2009). Depiction is one of these communicative devices often used along with conventional linguistic forms. When depicting, speakers can vocally represent an object or event in an iconic and meaningful way (Clark and Gerrig, 1990). The goal in depiction is to set up a physical scene that is analogous to the real-world scene, and to invite the listener to imagine the sensory or visual experience (Clark and Gerrig, 1990; Clark, 2016). The forms used in a depiction are often unconventional, and map onto meaning gradiently rather than categorically (Shintel et al., 2006). Users of sign language also make use of depiction (Liddell, 2003; Streeck, 2008), innovating visual forms that map gradiently onto meaning (Okrent, 2002; Emmorey and Herzig, 2003). Here we focus on how depiction is used by signers in situations designed to be difficult to describe.

### Depiction in Spoken Languages

Depiction in speakers can have a significant semantic, and possibly even grammatical role, in a sentence (Ferrara and Johnston, 2014; Hodge and Johnston, 2014). For example, a depiction can function as a noun or verb phrase, embedded within a larger phrase (Clark, 2016). In example (3), the speaker was discussing a piece played by Bela Bartok. He starts his sentence by saying, “he does not play,” and then depicts a musical passage on the piano using a style not used by Bartok—this passage takes on the role of a noun phrase in the speaker’s sentence. The speaker then contrasts this depiction with another depiction, a musical rendition of how Bartok *did* play the passage, which also takes on the role of a noun phrase.

(3) He does not play (*demonstrates four measures on the piano while singing*) but rather he plays (*demonstrates the same four measures while singing, but differently*)—and he does it better than I do. Clark (2016)

These musical depictions function as parts of the speaker’s sentence, and the speaker uses them contrastively to highlight how the piece was actually played. Note that the speaker’s sentence is actually incomplete without the two performative chunks. However, even though these depictions are functioning as part of the speaker’s sentence, they are not conventional linguistic forms. In the *Bartok* example (3), the depictive forms were created on the spot by the speaker, but can be immediately understood by others through the iconic mapping between the forms and the events they represent.

Speakers also use depiction to demonstrate the speech, affect, and emotions of another person. In the following example (4), Matt talks about a customer, Beth.

(4) She says ‘well, I’d like to buy an ant’ Clark and Gerrig (1990)

Matt is not referring to himself when he says “I,” but is instead taking on the role of Beth, who is talking to a store clerk. As such, he may also be raising the pitch of his voice and gesturing as Beth might gesture. Examples of this sort are referred to as role shift, direct quotation, direct speech, or constructed dialog.

Depictions can contain a mixture of categorical and gradient forms. Mimetic words, called ideophones, found in languages like Japanese or Siwu (Kita, 1997; Dingemanse and Akita, 2016), are good examples. These spoken devices are iconic, sensory words that contain properties amenable to gradient modification (e.g., reduplication or vowel lengthening). A Japanese speaker can reduplicate the ideophone *zorot(-te)*, which means ‘one after another in line,’ to create *zorozorot-te*, which iconically expresses the intensity of incoming waves. This kind of expressive foregrounding is not limited to mimetic linguistic forms. Non-mimetic words can be modified analogically by, for example, elongating the vowel “o” in the word *long* in a meaningful way: “It was a *loooooong* time” (Okrent, 2002; Shintel et al., 2006). The meaning of the categorical form is preserved, while the analog acoustic overlay contributes additional rich meaning (Kästner and Newen, 2017).

### Depiction in Sign Languages

Depiction is not only prevalent in everyday spoken language, but is also common in signed language. Signers often use constructed dialog or constructed action, where the signer takes on the role of another person or produces an action of another person or object (Metzger, 1995; Quinto-Pozos, 2007; Cormier et al., 2015). In Quinto-Pozos (2007) study eliciting utterances with constructed action, a signer takes on the role of a seal and enacts its behavior by using his body to represent the animal’s body. The hands form 5 (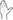) handshapes and are placed on the side of the body to represent the flippers. Then, the head and torso sway forward and back, and the mouth opens and closes as if representing the mouth of seal, which appears to be pure enactment.

In a different example, a signer first identifies an agent by signing WOMAN, and then describes the agent’s goal—MONEY HOW-MUCH TOTAL (5); the woman wanted to know the total cost (Cormier et al., 2012). The signer then enacts the woman’s actions on a calculator by using her body to represent the woman’s body and her hands to represent the woman’s hands on the calculator. The handling handshape representing the agent’s actions on the calculator is considered to be a depicting construction (DC). As in the *Bela Bartok* example above, the depiction completes the sentence and makes it comprehensible.

(5) WOMAN MONEY HOW-MUCH TOTAL (*enacts using the calculator with handling DC*) Cormier et al. (2012)

Signers also use DCs, also known as classifier constructions. DCs are comparable to Japanese mimetics in the sense that they are gradient and can represent physical properties of events. In these predicates, handshape is used to denote the category of the object being described; for example, an index finger (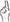) handshape represents a long, thin object, or a bent-V (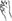) handshape represents an animal (Supalla, 1982, 1986). These forms are conventional, although they can take on mimetic properties (as do ideophones in speech); for example, the handshape can be iconically and topographically moved in sign space to portray the location and motion of the object. In these constructions, handshape is categorical, with clear and predicable mappings onto semantic categories, but movement is arguably gradient (Emmorey and Herzig, 2003; Liddell, 2003; although see Supalla, 1982, for a different view). This gradient property of DCs makes these forms highly productive; signers can combine multiple components and manipulate them gradiently in space in a seemingly infinite number of ways.

The sign language literature typically characterizes a linguistic form as *either* categorical *or* gradient. But it can, in fact, be both (Emmorey and Herzig, 2003). Recent research on Taiwanese Sign Language (e.g., Duncan, 2005) has shown that signers gradiently modify their categorical handshapes, often using these gradient devices to convey the same type of information that speakers incorporate into their co-speech gestures. Signers used an animal classifier handshape (thumb-and-pinky handshape) to represent the cat in a story, and gradiently modified the handshape to represent the cat’s ever-changing body form as it moved up the drainpipe. Indeed the signers used the same modifications to capture the cat’s movements that hearing speakers use in their co-speech gestures describing the same scene (Duncan, 2005).

### The Current Study

The goal of our paper is to characterize depiction in signers with an eye toward similar phenomena in spoken languages. In previous work, we developed a paradigm for eliciting depiction in speakers (Lu and Goldin-Meadow, 2017). Here we apply this paradigm to signers to explore depiction in the manual modality. In a within-subjects design, we asked speakers to describe two objects that could easily be characterized using lexical words in English (Descriptive Elicitation), and two objects that were more difficult to distinguish using English lexical words, thus encouraging speakers to depict (Depiction Elicitation). When speakers struggle to access lexical words, they gesture at relatively high rates (Chawla and Krauss, 1994; Hostetter et al., 2007; Sevcikova Sehyr et al., 2018), and we found that speakers did indeed use more iconic gestures in the Depictive Elicitation context than in the Descriptive Elicitation context (Lu and Goldin-Meadow, 2017).

Here we ask how signers behave in Depictive Elicitation contexts, and focus on the depiction of the size and shape of objects, where handshape and movement contribute to creating meaning about both properties. Depiction of size and shape is a relatively underexplored area compared to depiction of action, handling, or viewpoint (Metzger, 1995; Quinto-Pozos, 2007; Cormier et al., 2015). Previous literature has characterized handshape and movement within DCs as categorical (e.g., Supalla, 1986); we extend this research by exploring other kinds of DCs that have imagistic and gradient qualities.

We developed a paradigm that systematically elicits depictive devices. Signers described to a camera pairs of objects that belonged to the same category and were of the same color but differed in shape (e.g., a yellow vase of shape 1 vs. yellow vase of shape 2). The lexical signs YELLOW and VASE do not distinguish between the two vases, and there are no lexical items in ASL that correspond to the difference in shape between the two yellow vases. As a result, signers may feel the need to depict. We explore the depictive strategies signers use when lexical signs alone are not likely to suffice in communicating the full message.

## Materials and Methods

### Participants

Nineteen deaf participants, fluent in American Sign Language (ASL), were recruited at a local Deaf event or through email advertisement. Four participants were excluded from the data analyses because they did not understand the instructions (e.g., they created elaborate stories unrelated to the task, or did not use any lexical signs in their descriptions; *n* = 3) or because they had proficiency in another sign language prior to learning ASL (*n* = 1). Data from the remaining 15 participants were coded by a deaf and a hearing coder, both of whom were fluent in ASL. The mean age of first exposure to ASL is 0.43 years (*SD* = 0.82, range: 0–3 years), and 10 out of 15 participants were native signers born to deaf parents. Thirteen participants gave ASL as their preferred language; the remaining two did not respond to the question. Signers were paid $50 for their participation and travel.

**FIGURE 1:**
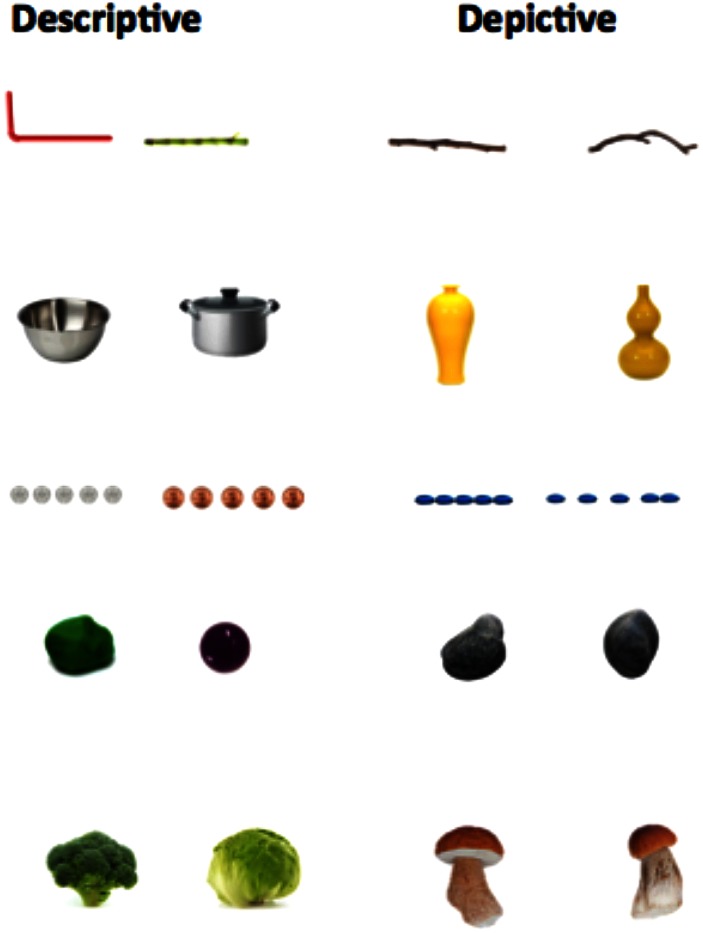
Pairs of stimuli presented in Descriptive and Depictive Contexts.

The stimuli were 24 pairs of objects presented on a computer screen, 12 in the Depictive Elicitation context (pairs of objects that are difficult to distinguish using lexical signs, e.g., a yellow vase of shape 1 vs. a yellow vase of shape 2; **Figure 1**; Supplementary Material), and 12 in the Descriptive Elicitation context (pairs of objects that could be identified by different lexical signs, e.g., pot vs. bowl)^1^. The presentation of stimuli was programmed on Psyscope X B77 (Cohen et al., 1993). The objects in both the Descriptive and Depictive Elicitation contexts belonged to five different shape categories: (1) long and thin objects (e.g., a stick), (2) small and discrete objects (e.g., pills), (3) cylindrical objects (e.g., a vase), (4) round objects (e.g., a rock), and (5) objects with a combination of shapes, (e.g., a mushroom, which was a combination of a thin stem and a round cap). The contexts were designed to elicit the following handshapes—Claw 5 (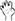), C (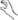), F (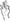) handshapes (see Supplementary Tables S1, S2). The position of the objects appearing on the left or right side of the screen was counterbalanced, and the order of presentation of stimuli was random.

### Procedure

Prior to the study, participants were interviewed online about their language and education background, and also filled out consent forms and a questionnaire on their language background. Deaf participants were asked in ASL by a native deaf signer to describe what they saw on the screen; they were told that this was a communication task and that another participant would later watch the video of their responses and be asked to identify which of the two objects the response referred to. Once they completed their responses, the experimenter debriefed the participants on the goal of the study.

### Coding

**FIGURE 2:**
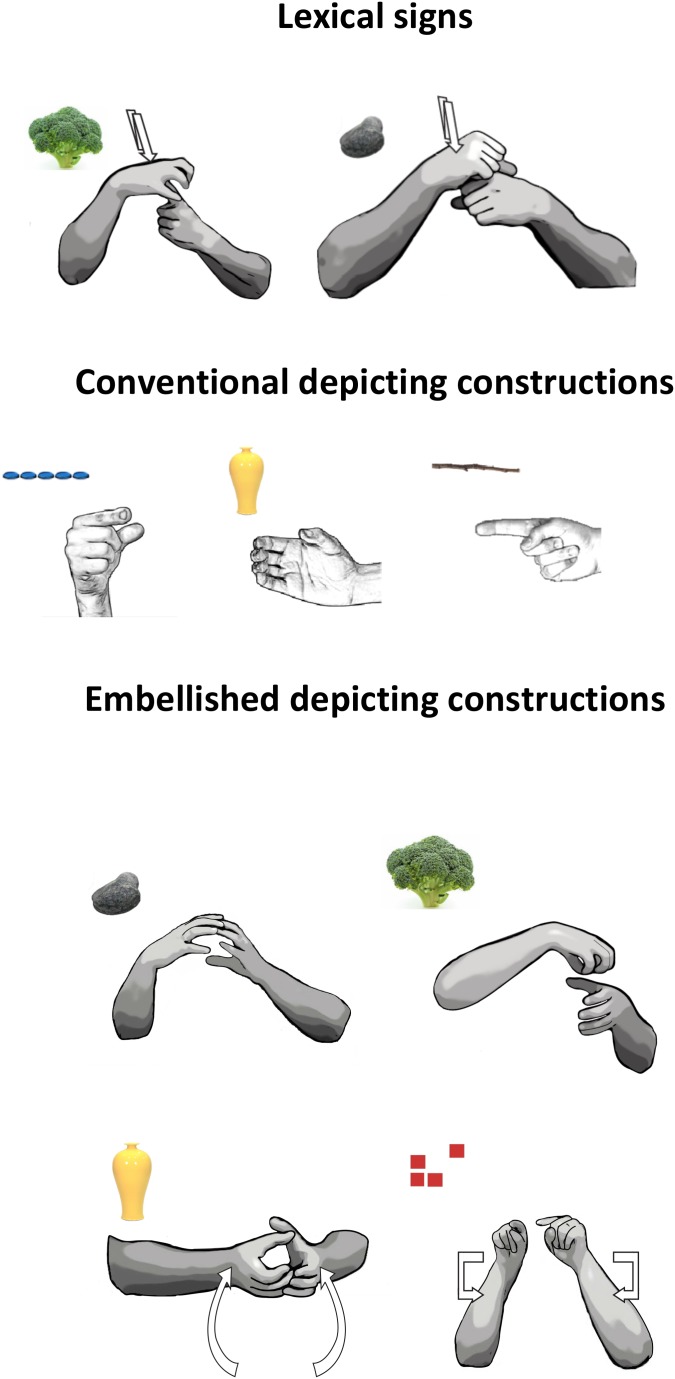
Examples of lexical signs, conventional DCs, and embellished DCs. **(Top)** Lexical signs for BROCCOLI and ROCK. **(Middle)** Conventional DCs used to represent a small and discrete (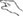) object (left), like a pill; a long and thin (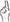) object, like a stick (middle); and a cylindrical (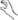) object, like a vase (right). *Third row*: two-handed embellished DCs used to depict the shape of a rock with two Claw-5 (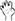) handshapes (left), and the shape of a broccoli with a C (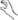) handshape capturing the stem of the broccoli and a Claw-5 (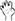) handshape capturing its florets (right). **(Bottom)** Tracing embellished DCs used to depict the shape of a vase (a 3D representation) with two Claw-5 (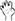) handshapes (left), and the outline of a square (a 2D representation) with index fingers (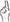) (right).

We transcribed all of the lexical items and DCs that each participant produced in the Depictive and Descriptive Elicitation contexts. We included in the analyses core lexical signs (Brentari and Padden, 2001) and fingerspelled words serving as nouns or adjectives, as well as DCs describing perceptual attributes of the objects (see **Figure 2**). In a lexical sign, the handshape, location, and movement are fixed (unless inflected by a regular morphological process).

Depicting constructions are also called “classifiers” (Frishberg, 1975; Kegl and Wilbur, 1976; Supalla, 1986), “polymorphemic signs” (Engberg-Pederson, 1993), “polycomponential signs” (Slobin et al., 2003), and “depicting verbs” (Liddell, 2003). Following Johnston and Schembri (2010) and Cormier et al. (2012), we use the term “depicting constructions,” DCs. Each component of a DC—handshape, location, movement—bears meaning (unlike phonological components of signs), and each is a bound morpheme that can recombine with each other. We excluded the few DCs that were used to describe actions performed on or with the objects (e.g., handling DCs), as well as pointing and numbers signs. We identified two main types of DCs in our data: conventional DCs and embellished DCs.

#### Conventional DCs

These DCs are also known as “size and shape specifiers” (SASS) or, more recently, “entity DCs” (Cormier et al., 2012; Zwitserlood, 2012). The handshape in these DCs represents the shape of the object (Supalla, 1986). For example, a signer could use an F (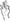) handshape to represent a coin, a C (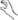) handshape to represent a bottle, or an index finger (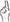) handshape to represent a pen. The handshapes in conventional DC’s are fixed but, unlike lexical signs, can combine with a variety of movements or locations. However, the handshapes in the conventional DCs produced in our study were combined, for the most part, with a hold movement, or a series of holds, in neutral space. If the signer produced a series of repeated handshapes, without pausing, to indicate a set of items (e.g., pills), this response was coded as a single conventional DC. If the signer described the first three pills in a row, paused, and then described the second two pills (which were spaced closer together than the other three), this response was coded as two separate conventional DCs (see **Figure 2**).

#### Embellished DCs

Participants also produced DCs that have imagistic components added to a conventional base. Conventional DCs draw from a conventional set of handshapes (Supalla, 1986; Zwitserlood, 2003). Embellished DCs use these same handshapes but embellish them, either by adding a second conventional handshape or by adding movement. These embellished DCs appear to be spontaneously created at the moment to capture particular aspects of the objects to which they refer.

##### Combining two handshapes

Combining two conventional handshapes allows signers to capture objects with a complex configuration or with multiple parts (see Sevcikova Sehyr et al., 2018). For example, a signer can use a C (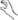) handshape on the non-dominant hand to represent the stem of a broccoli and a Claw-5 (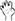) handshape on the dominant hand on top of the C (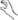) handshape to represent the florets (**Figure 2**). Two-handed DCs do not always contain two different handshapes; e.g., two of the same handshapes (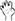) can combine to form a round configuration like a rock (**Figure 2**).

##### Adding a tracing movement

Adding movement to a conventional handshape allows signers to capture the object’s shape (see **Figure 2**). This type of sign, which traces the outline of the object in 3D space, has been called a “tracing SASS” (Supalla, 1982), a “contour sign” (Zwitserlood, 2003), “molding,” or “sculpting” where the hands “shape a transient sculpture in space” (Müller, 2013; see also Kendon, 2004; Nyst, 2016). For example, a signer moves two Claw-5 (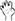) handshapes in and out while going from bottom to top in space in order to sculpt the outline of a vase. At times, signers used an index finger (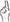), rather than a classifier handshape, to sketch or draw an object’s contour (Mandel, 1977; Müller, 1998; Müller, 2013; Nyst, 2016). Both of these types of tracing DCs can be performed either with one hand or with two identical hands (see **Figure 2**).

The two embellishing strategies that the signers used in our data to modify their handshapes both mimetically depicted aspects of the stimuli. However, the strategies lent themselves to capturing different features and thus were often used for different stimuli. The signers tended to add movement to depict long, thin objects and cylindrical objects; to add a second handshape to depict small, discrete objects; and to use both strategies (at approximately the same rates) to depict round objects and objects with a combination of shapes. We combined these strategies into a single category, which we called *Embellished DCs*.

In a few cases, there were challenges in distinguishing DCs from lexical signs that may have originated as DCs. For example, the sign for BOTTLE resembles a tracing DC and presumably was derived, at some point, from this spontaneous construction (Cormier et al., 2012). These ambiguous signs were relatively rare (99/2004 = 0.05 of all observations) and were excluded from the analyses.

In addition to depicting on the hands, signers also produced movements with their mouths that captured aspects of the objects, often the same aspects captured by the hands (see Sutton-Spence and Boyes Braem, 2001; Sandler, 2009). Using an expanded version of Anderson and Reilly’s (1998) coding system for mouth movements (e.g., glosses such as *ps* to indicate cheeks sucking in or *puff* to indicate puffed cheeks), we identified three types of mouth movements: mouthing, lexical mouth components, and iconic mouth movements.

#### Mouthing

Mouthing lexical words that are borrowed from spoken language (Sutton-Spence and Boyes Braem, 2001; Crasborn et al., 2008); e.g., mouthing the word “bottle” while producing the lexical sign for BOTTLE).

#### Lexical Mouth Components

Mouth movements that obligatorily co-occur with specific lexical items, but are not derived from speech (Meir and Sandler, 2008; Sandler, 2009); e.g., in Israeli Sign Language, a mouth movement “fa” has to be obligatorily produced with a sign meaning THE-REAL-THING; “fa” has no relation to the words in Hebrew that mean ‘the real thing.’

#### Iconic Mouth Movements

Mouth movements that depict the size and shape of the objects. These movements often capture aspects of the object that are simultaneously captured on the hands; e.g., puffing one’s cheeks three times as the hands trace the three bulges of the vase. This category includes mouth movements that Sandler (2009) categorized as adverbial or adjectival modification. However, in our study, signers rarely used a single adjectival mouth morpheme to modify a nominal sign, as in puffed cheeks used to mean big (Liddell, 1980; Sutton-Spence and Woll, 1999; Crasborn et al., 2008). In our study, signers typically produced sequences of mouth movements (rather than a single movement) to highlight a property of the object (presumably because of the types of objects we presented).

##### Reliability

A second coder, a hearing signer fluent in ASL, coded 20% of the participants to establish reliability. The coders agreed on 83% of decisions categorizing DCs and 80% of decisions categorizing mouth movements. Coders discussed their disagreements and reached full consensus on the categories.

## Results

### Signs Produced in Depictive vs. Descriptive Elicitation Contexts

**FIGURE 3:**
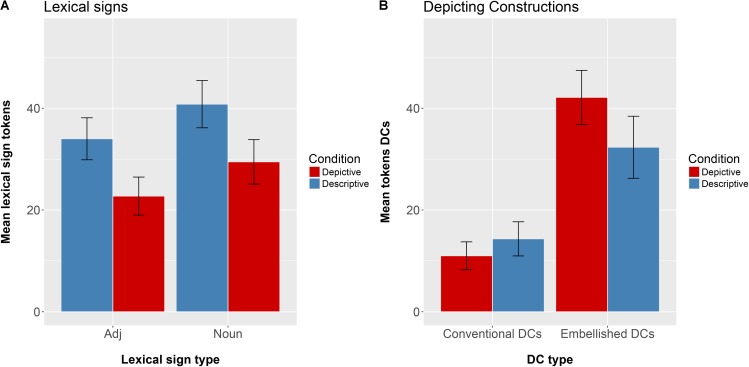
**(A)** Depicts the mean number of Adjectives and Nouns produced in the Descriptive Elicitation (blue) and the Depictive Elicitation (red) conditions. **(B)** Depicts the mean number of Conventional DCs and Embellished DCs produced in the Descriptive Elicitation (blue) and the Depictive Elicitation (red) conditions.

**Figure 3A** presents the mean number of lexical signs (adjectives and nouns) and **Figure 3B** presents the mean number of DCs (conventional and embellished) produced by our participants in the Descriptive Elicitation condition and in the Depictive Elicitation condition.

We first analyzed the patterns of lexical signs produced in Descriptive vs. Depiction conditions. We performed a 2 (Condition: Descriptive vs. Depictive) × 2 (Word type: Nouns vs. Adjectives) repeated measures ANOVA, using count of lexical signs as the dependent variable. As expected, there was a significant main effect of Condition, where participants produced more lexical items or fingerspelled words, either nouns (e.g., ‘bottle’ and ‘vase’) or adjectives (e.g., ‘thin’ and ‘yellow’), to describe the pairs of objects in the Descriptive Elicitation condition than in the Depictive Elicitation condition [*F*(1,14) = 51.13, *p* < 0.0005]. There was also a significant main effect of Word type, where subjects produced more nouns than adjectives [*F*(1,14) = 8.99, *p* < 0.005]. Finally, there was no significant interaction between Condition and Word type [*F*(1,14) = 0.0002, *p* = 0.90].

Next, we analyzed the patterns of DCs produced in Descriptive vs. Depiction conditions. We performed a 2 (Condition: Descriptive vs. Depiction) × 2 (Conventional DCs vs. Embellished DCs) repeated measures ANOVA, using count of DCs as the dependent variable. There was a significant main effect of Condition, where subjects produced more DCs in the Depictive Elicitation condition than in the Descriptive Elicitation condition [*F*(1,28) = 1.26, *p* < 0.05]. There was also a significant main effect of DC type, where participants produced more embellished DCs than conventional DCs [*F*(1,14) = 22.57, *p* < 0.0005]. There was also a significant interaction between Condition and DC type [*F*(1,28) = 8.54, *p* < 0.005]. We investigated this interaction further with *post hoc* tests, and found that, at an alpha level of 0.025, signers produced significantly more embellished DCs in the Depictive Elicitation condition than in the Descriptive Elicitation condition (*p* < 0.005). In contrast, the number of conventional DCs that the participants produced did not vary by condition (*p* = 0.05). Embellished DCs have better potential to mimetically capture the size and shape of the objects than conventional DCs. The signers took advantage of this potential and used more embellished DCs in the Depictive condition than in the Descriptive condition. In contrast, they used the same number of conventional DCs in the two conditions, underscoring the depictive limitations of this type of DC.

Our paradigm was thus successful in eliciting depiction in signers. We focus for the remainder of this paper on DCs produced in the Depictive Elicitation condition.

### Conventional vs. Embellished DCs

**Table 1** presents the total number of conventional DCs and embellished DCs produced by each participant. Note that all 15 participants produced instances of each type of DC.

**Table 1:** Total number of Conventional and Embellished DCs produced by each participant in the Descriptive and Depictive Elicitation Conditions.

	Descriptive condition		Depictive condition	
	
Participant	Conventional	Embellished	Conventional	Embellished
	DCs	DCs	DCs	DCs
1	12	14	10	19
2	1	7	8	16
3	8	21	33	23
4	2	14	6	28
5	12	14	9	42
6	31	23	5	27
7	3	14	3	30
8	15	26	6	41
9	10	33	9	42
10	40	58	5	51
11	0	12	3	40
12	24	56	10	51
13	5	60	5	58
14	37	46	39	73
15	15	87	14	91

We explored whether signers used different depictive strategies depending on the stimulus type. As we saw in **Figure 1**, the objects fell into five different shape categories: (1) long and thin objects (e.g., a stick), (2) small and discrete objects (e.g., pills), (3) cylindrical objects (e.g., a vase), (4) round objects (e.g., a rock), and (5) objects with a combination of shapes (e.g., a mushroom which has both a round part and a long thin part). We selected one representative stimulus from each shape category, and analyzed the total number of conventional vs. embellished DCs that each participant produced (see **Table 2**). Signers used conventional DCs primarily for the small and discrete objects, and embellished DCs for all of the other objects.

**Table 2:** Number of conventional and embellished DCs produced by each participant for five representative stimuli.

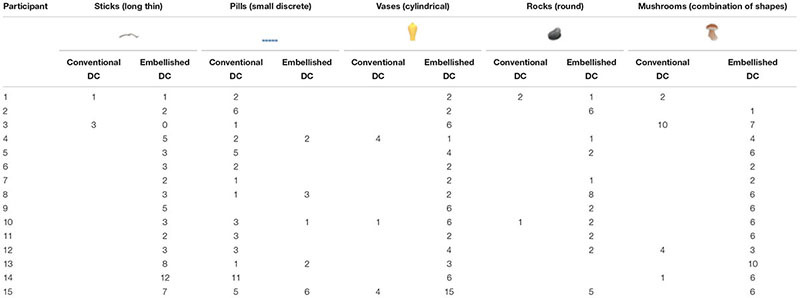

*For each stimulus, we counted the total number of conventional DCs and embellished DCs the participant produced for each pair of objects (e.g., Stick 1 and Stick 2; Vase 1 and Vase 2)*.

**FIGURE 4:**
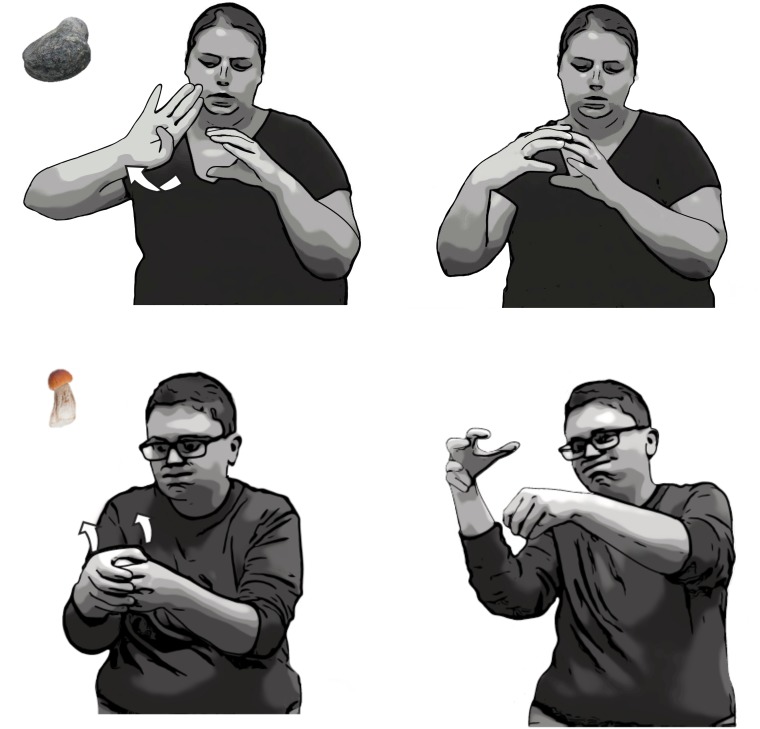
Examples of a combination of embellished DCs. **(Top)** In the image on the left, the non-dominant hand forms a Claw-5 (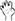) handshape, while the B (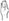) handshape on the dominant hand produces an embellished DC (in which she adds a tracing movement to capture the curvature of the rock). In the right image, the signer ends with another embellished DC (in which she adds a second handshape) to depict the whole shape of the rock. **(Bottom)** To depict a mushroom, the signer first adds a tracing movement to two C (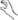) handshapes to depict the stem, and then combines two handshapes, the non-dominant C (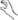) handshapes to depict the stem and the dominant Claw-5 (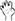) handshape to depict the mushroom cap.

Signers also produced a combination of DCs to describe a single object. For example, to describe a rock, a signer first produced a Claw-5 (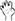) handshape with her left hand, and then, with her right hand in a B (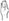) handshape, traced a curved trajectory on the Claw-5 (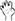) and thus produced an embellished DC. She ended by producing a second embellished DC — two Claw-5 (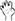) handshapes touching at the fingertips representing the rock as a whole (**Figure 4**, top row). This sequence could be described as a decomposed depiction, where the parts of the object are described sequentially (Sowa and Wachsmuth, 2009). The sequence contains a “frame hold” in which the non-dominant hand remains still while the dominant hand performs the tracing depiction (the embellished DC).

As an example of a combination of embellished DCs used to describe a mushroom, one signer first added a tracing movement to two C (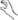) handshapes to depict the stem, and then combined two handshapes—the non-dominant C (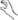) handshape depicting the stem and the dominant Claw-5 (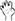) hand depicting the mushroom cap (**Figure 4**, bottom row). This is an example of a decomposed depiction where the signer uses two forms of embellished DCs to form an entire image of the object.

### Mouth Movements in the Depictive Eliciting Condition

Signers used mouth movements with over half of their lexical signs (*M* = 55%, *SD* = 15%). They also used mouth movements with about a third of their Embellished DCs (*M* = 36%, *SD* = 12%), but used them with very few of their Conventional DCs (*M* = 8%, *SD* = 5%). We found a significant effect of sign type on the number of mouth movements that accompanied the signs [*F*(2,28) = 25.01, *p* < 0.0001]. Lexical signs were significantly more likely to be produced with mouth movements than both types of DCs (embellished DCs, *p* < 0.01; conventional DCs, *p* < 0.01), and embellished DCs were significantly more likely to be produced with mouth movements than conventional DCs (*p* < 0.01).

**FIGURE 5:**
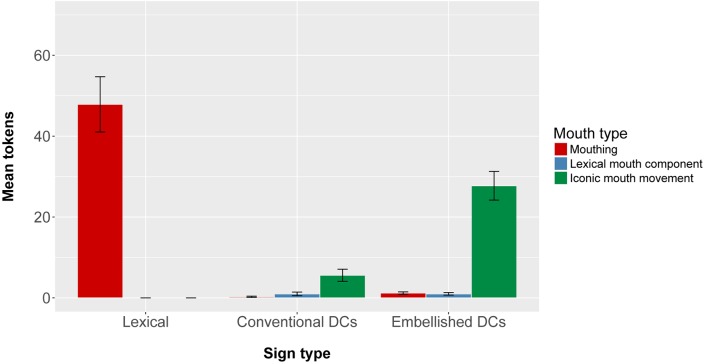
Mean number of Lexical Signs, Conventional DCs, and Embellished DCs produced by participants in the Depictive Elicitation Condition, classified according to whether they co-occurred with Mouthings, Lexical mouth movements, or Iconic mouth movements.

Moreover, signers used different mouth movements with each sign type. **Figure 5** presents the mean number of tokens of lexical signs, conventional DCs, and embellished DCs that co-occurred with iconic mouth movements (green bars), mouthing (red bars), and lexical mouth movements (blue bars). We ran a 3x3 repeated measures ANOVA (Sign type: Conventional DCs, Embellished DCs, and Lexical Signs) × Mouth Movement type (Iconic mouth movements, Mouthing, Lexical mouth components) using count of signs as the dependent variable. We found a significant main effect of Mouth Movement type [*F*(2,28) = 26.45, *p* < 0.0005], where signers produced significantly more lexical mouthings (*p* < 0.005) and significantly more iconic mouth movements (*p* < 0.005) than lexical mouth components. We also found a significant main effect of Sign type [*F*(2,28) = 24.99, *p* < 0.0005], where signers produced significantly more lexical signs than conventional DCs (*p* < 0.005); and significantly more embellished DCs than conventional DCs (*p* < 0.005).

Importantly, there was a significant interaction between Mouth Movement type and Sign type [*F*(4,56) = 61.71, *p* < 0.0005]. We followed up this interaction with *post hoc* tests at the alpha level of 0.017. Mouthings appeared more often with lexical signs than with either conventional DCs (*p* < 0.0005) or embellished DCs (*p* < 0.0005), and more often with conventional DCs than embellished DCs (*p* < 0.05). In contrast, iconic mouth movements appeared significantly more often with DCs (both embellished, *p* < 0.0005, and conventional, *p* < 0.05) than with lexical signs, and more often with embellished DCs than with conventional DCs (*p* < 0.0005). Lexical mouth components were rarely produced with any of the three sign types. The fact that iconic mouth movements co-occurred most often with embellished DCs underscores the imagistic aspect of these depictive signs.

#### Iconic Mouth Movements Produced With Embellished DCs

**FIGURE 6:**
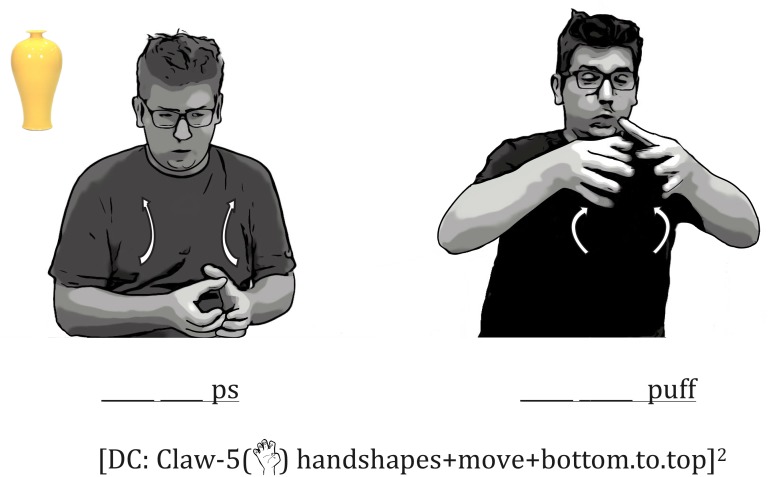
An Example of a Series of Iconic Mouth Movements Combined with an Embellished DC. The signer started off with hands close together to indicate the narrow part of the vase while sucking in his cheeks, and then widened the distance between his hands while puffing his cheeks. The hands and mouth movements are thus tightly correlated in size and shape.

Signers frequently exploited the same iconic mapping in their iconic mouth movements that they displayed in their embellished DCs. For example, one signer sucked in his cheeks (a *ps* mouth movement), which evokes an imagery of thinness, while at the same time tracing the bottom, thinner part of the vase with two Claw-5 (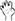) handshapes (the hands were held close together in space). The mouth then transitioned to *puffed cheeks* while the hands traced the top, wider part of the vase (the distance between the hands increased (**Figure 6**)^2^. The change from one mouth shape to another is gradual, and is tightly correlated with the changes in the space between the two hands in the embellished DC.

**FIGURE 7:**
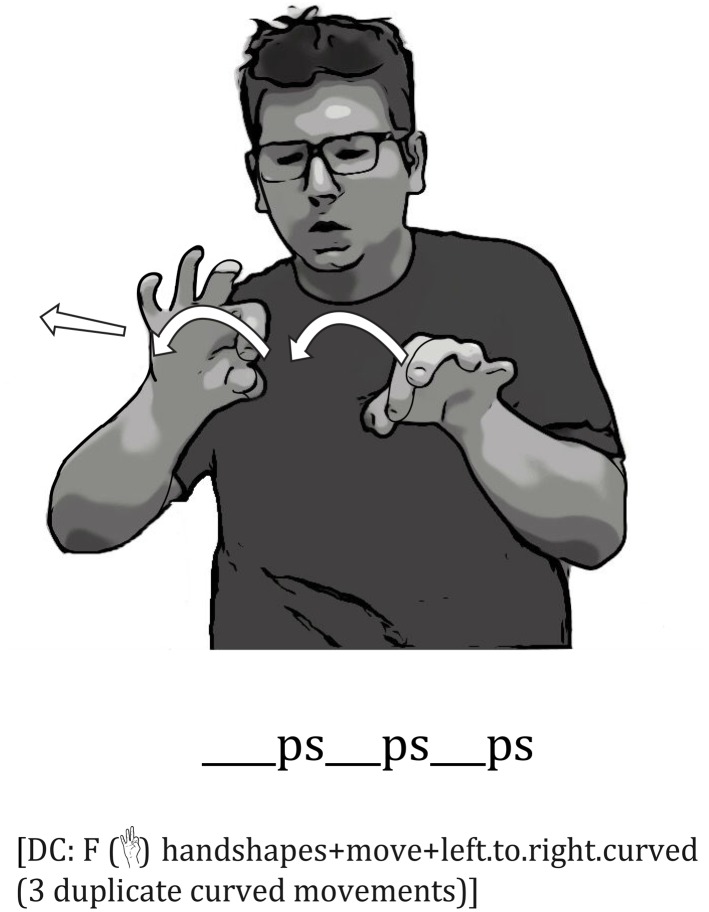
An Example of Reduplicated Iconic Mouth Movements Combined with an Embellished DC. The signer produced the mouth shape, *ps*, three times while tracing the three curves of the stick with a (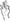) handshape, thus displaying a perfect correspondence between the individual *ps* movements and each curve in the embellished DC.

Not only do signers gradiently modify their mouth shapes, transitioning from one mouth shape to another, but they often reduplicate the same mouth shape to reflect repeated properties of the object. These mouth reduplications correspond to the spatial reduplications of tracing movements in the embellished DCs. For example, in describing a tree branch with three curves, one signer reduplicated his mouth shape, *ps*, three times as he traced the three curves with his dominant hand in a G (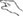) handshape, thus displaying a perfect correspondence between his mouth movements and the hand in his embellished DC (**Figure 7**).

**FIGURE 8:**
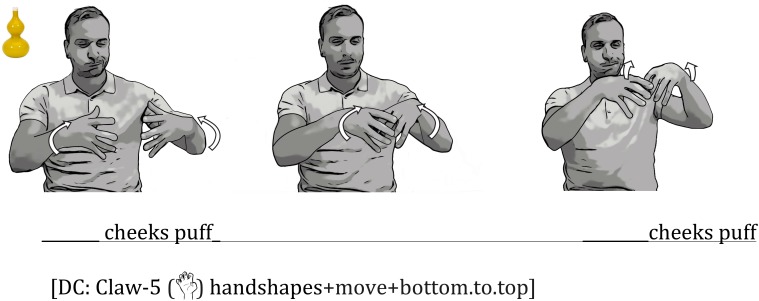
An Example of Iconic Mouth Movements Produced with an Embellished DC. The signer produced two cheek puffs, one as he sculpted the bottom bulge of the vase and one as he sculpted the top bulge.

In another example of reduplicated mouth movements combined with an embellished DC, a signer produced two cheek puffs while tracing the two round parts of a yellow vase (**Figure 8**). The signer puffed his cheeks while first tracing the bottom round part of the vase; as he sculpted the middle part of the vase with his hands, he shrunk his cheek puffs; finally, as he traced the top round part of the vase with his hand, he puffed his cheeks again.

#### Iconic Mouth Movements Produced With Conventional DCs

**FIGURE 9:**
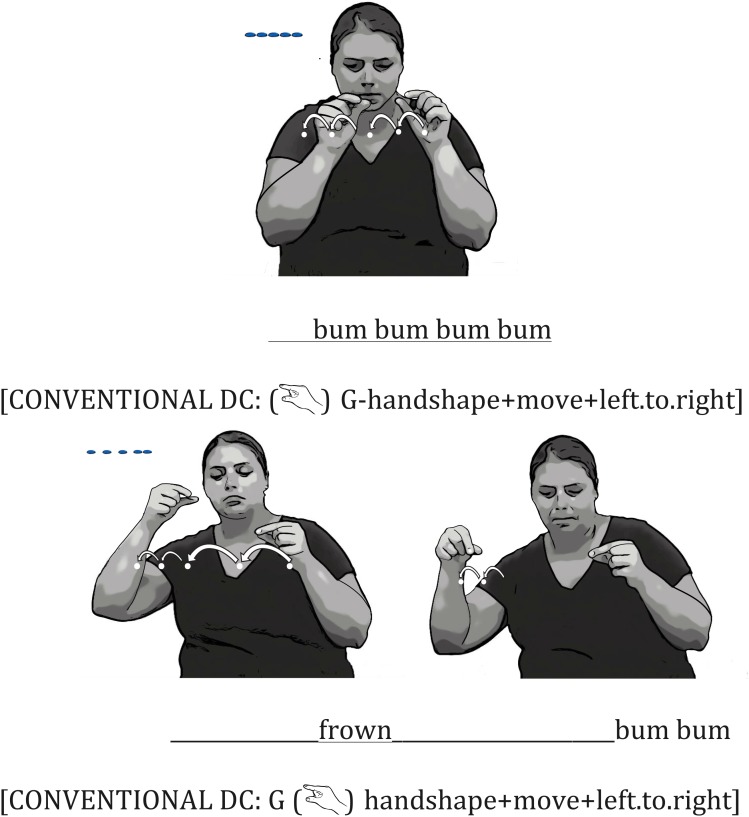
An Example of an Iconic Mouth Movement Combined with a Conventional DC. The signer used a G (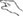) handshape to represent each individual pill in both descriptions. To describe the first array in which five pills were evenly spaced **(top)**, she produced a repetitive *bum* mouth movement as she laid out each pill with her hands; the spacing between each pair of pills was even in both the signer’s mouth and hand movements. To describe the second array in which three pills were evenly spaced and separated from two pills, which were more closely spaced **(bottom)**, she produced a *frown* mouth movement as she placed the first three pills, and then a *bum bum* mouth movements as she placed the last two pills.

Conventional DCs were often produced to describe the smaller objects, particularly those laid out in distinct arrays. For example, to describe two arrays of pills (see **Figure 1**), one signer used a conventional DC handshape (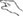) to represent each individual pill, and laid the set of DCs out in space. Note that signing space is used differently in conventional DCs than in embellished DCs. In embellished DCs, space is used to represent the shape of a single object, but in conventional DCs, space is used to represent an arrangement of multiple objects. The signer also varied her mouth movements to capture the distance between the pills. For the first set of five pills, where the pills were evenly spaced, the signer produced a repetitive *bum* mouth movement as she laid out each pill. For the second set of five pills, where the first three were evenly spaced and separated from the second 2 (which were closely spaced), the signer produced a *frown* mouth movement to capture the relatively wide distance between the pills for the first three pills, and then a repetitive *bum bum* mouth movement to capture the closer distance between the last two pills (see **Figure 9**). Iconic mouth movements co-vary with hand movements in conventional DCs, as they do in embellished DCs.

### Individual Differences in Depicting

**FIGURE 10:**
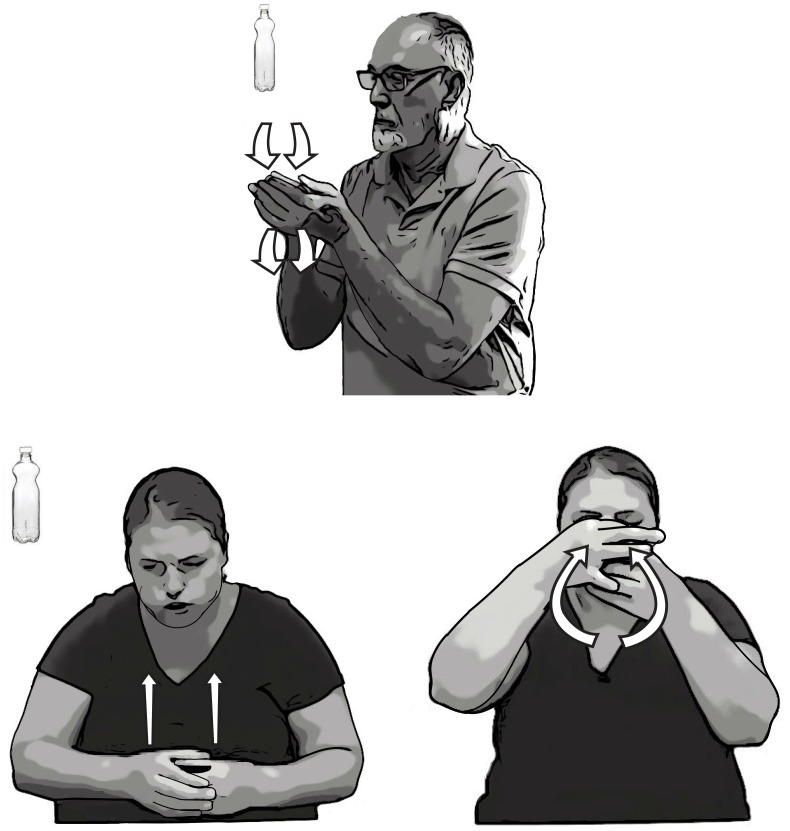
An example of individual variation in the embellished DCs signers use to portray a water bottle. The signer in the top image traced the top of the bottle with two bumps with 5 (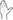) handshapes. The signer in the bottom images traced the bottle from bottom to top with C (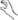) handshapes and then transitioned to an F (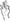) handshapes at the top.

Signers varied in the particular movements they produced in their embellished DCs. These variations suggest that embellished DCs were likely to have been created in the moment rather than drawn from a conventional store of movements. Some signers were more veridical to the size and shape of the objects they described, and traced the entire object. Other signers were less specific and captured only the distinguishing features of the objects in their tracings. For example, one signer was attentive to the details of the shape of a water bottle in her description of the bottle and traced its entire contour, depicting the narrower circumference of the bottom half of the bottle and the two bumps on the top (**Figure 10**, bottom row). Another signer did not trace the bottom of the bottle and represented only the two bumps on the top in her tracing DC (**Figure 10**, top). Additionally, these two signers used slightly different handshapes—one signer used 5 (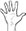) handshapes on both hands (**Figure 10**, top) and the other signer used C (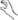) handshapes and then transitioned to F (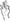) handshapes as she traced the top of the bottle (**Figure 10**, bottom).

Signers also varied in how they combined the two handshapes in their embellished DCs. For example, in depicting a rock, some signers produced a combination of DCs by holding a Claw-5 (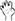) handshape on the left, and then using a B (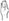) handshape on the right hand to trace the curvature (**Figure 4**). Other signers would instead hold a Claw-5 (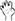) handshape on the left, and then place another Claw-5 (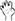) handshape on the top and not use any tracing movement.

**FIGURE 11:**
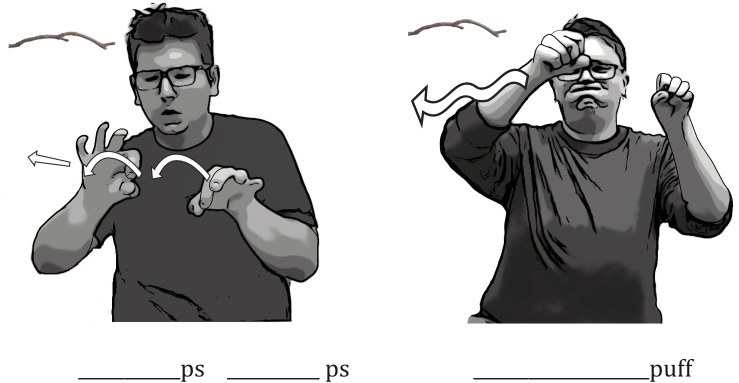
An example of individual variation in the embellished DCs and iconic mouth movements signers used to portray a branch. The signer on the left traced the branch with an F (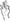) handshape while reduplicating the *ps* mouth movement. The signer on the right traced the branch with two S (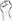) handshapes while producing one continuous cheek-puffing mouth movement.

The variation across individuals in hand tracings and hand combinations was paralleled by variation in iconic mouth movements. To describe a stick, one signer traced the branch with an F (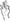) handshape and reduplicated the *ps* mouth shape (**Figure 11**). To describe the same stick, another signer used two S (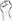) handshapes that were slightly open to trace the curve of the stick, and produced one continuous mouth movement by puffing her cheeks and bottom lip.

In contrast to the variability found in embellished DCs and iconic mouth movements, there was (not surprisingly) less variability across signers in the conventional DCs. Signers tended to use the same handshape to represent a particular shape, for example, a G (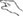) handshape for small and discrete items, and a C (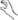) handshape to represent cylindrical objects, each with a hold movement. Moreover, they used relatively few mouth movements with conventional DCs and, when they did use a mouth movement with this type of sign, they tended to draw from a relatively small set of mouth movements (e.g., usually single cheek puffs).

## Discussion

Signers use multiple strategies to depict the size and shape of objects. When it is difficult to succinctly distinguish between two objects using lexical labels, signers resort to depiction. They recruit conventional and embellished DCs, and combine each type with iconic mouth movements to imagistically capture the sizes and shapes of the objects they are describing. However, embellished DCs occurred more often in Depictive contexts than in Descriptive contexts, whereas conventional DCs occurred equally often in the two contexts. Moreover, embellished DCs co-occurred more frequently with iconic mouth movements than conventional DCs, and may be more tightly integrated, temporally and spatially, with these mouth movements than conventional DCs. Taken together, these findings suggest that embellished DCs share properties with co-speech gestures in that both are imagistic and spontaneously created.

### Depicting Constructions vs. Lexical Signs

Depicting constructions are a heavily used resource for signers to describe the size and shape of objects. Signers often choose to depict rather than use available lexical signs, such as LONG, TALL, or MIDDLE. Interestingly, when asked to distinguish between the same pairs of objects, speakers often use a litany of adjectives and invoke specific scenarios (e.g., “a vase that you can put a flower in”; Lu and Goldin-Meadow, 2017). In one example (6), an English speaker said the following to distinguish between the two yellow vases in **Figure 1**:

(6) “One of the objects in this one is a yellow... Looks like a vase that you can put a flower in. Um it’s like it gets *slimmer* as you go toward the *bottom* whereas the other object could also be a flower vase, there are like two different bumps in the *middle* and at the *bottom*.”

The speaker also produced many iconic co-speech gestures along with his many adjectives, *slim*, *middle*, and *bottom*, to describe different aspects of the vase. Signers rarely used the sign equivalents of these adjectives, even though they are available in ASL. They relied entirely on depictive devices to convey size and shape.

Why might it be easier to depict in sign rather than use descriptive lexical items, and what prompts signers to rely on these depictive devices? We can imagine several factors that could lead to heavy use of depiction in sign: (1) It may be particularly efficient to use DCs, which can encode two characteristics of an object (e.g., width and height) simultaneously within a single construction; encoding the same information lexically would require several signs (Quinto-Pozos, 2007; Sevcikova Sehyr et al., 2018). (2) Many lexical signs in ASL have an iconic base, and these iconic properties might clash with the meaning of the objects being described (cf. Meir, 2010); using a depictive device would circumvent this potential difficulty. For example, the sign THIN involves using two pinky fingers that first contact each other and then move in opposite directions vertically in space. The vertical movement of this lexical form nicely captures objects that are thin and upright, such as a candle. Signers are likely to use this lexical sign in this case. However, this form is a less good rendition of objects that are thin and *horizontal*, such as the sticks in our study. Signers may therefore be less inclined to use the lexical sign THIN, which is produced vertically in space, to describe a horizontal stick. Instead, they turn to an embellished DC, tracing a G (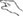) handshape horizontally in space. When lexical signs do not map neatly onto their referents, signers may choose to depict using embellished DCs so that they can be faithful to the iconic mapping. Future work is needed test this hypothesis by exploring whether lexical signs are more likely to be used than depictions when they can be fully mapped onto the form of their referents. (3) ASL signers and English speakers have different lexicons (Quinto-Pozos, 2007; Sevcikova Sehyr et al., 2018), and some of the stimuli in our study were difficult to label with a single lexical sign in ASL (e.g., *knob* of the French press; *florets* of the broccoli; *tip* of the screwdriver). Signers may need to resort to depiction, using embellished DCs in particular, to describe items that English speakers can reference with lexical labels (there are, of course, items that do not have ready labels in English, and we predict that English-speakers will rely on depiction for these items).

### Embedded Depiction in Signed and Spoken Languages

Depicting constructions play an important role within a signed utterances in that, if they are removed, significant information is lost. DCs can be analyzed as a constituent within a clause-like unit (Ferrara and Johnston, 2014; Hodge and Johnston, 2014), just as embedded depictions can be analyzed as linguistic units in spoken languages (Clark, 2016). In the *Bartok* example presented earlier (2), the pianist starts his sentence with, “he does not play,” and then depicts what the pianist doesn’t do by playing a short Mozart passage, a depiction that functions as a noun phrase.

In our data, signers often began by first naming the object (e.g., using the lexical sign for vase) and then following that description with a spontaneously created depiction (e.g., tracing the contour of the vase; using two hands to indicate the configuration of the vase). These depictions thus function like adjectival predicates. Similar structures can be found in speech. For example, a speaker begins by describing the vase (“there is a vase”), and then switches into depiction by tracing the two bumps of the vase in a co-speech gesture; gestures of this sort are often accompanied by sound effects (in this case, *bum bum*), which seem to function like iconic mouth movements. As in sign, this depiction serves as an adjectival predicate, and the shape information is not found anywhere else within the clause.

These depictions in speech and sign contain gestural materials in the sense that the forms are not conventional, and are unlikely to have been drawn from a lexicon of words or signs. Nevertheless, the forms often take on the full weight of expressing information about the size and shape of an object (which is not conveyed anywhere else in the utterance). The gestural materials work together with the linguistic materials to form a composite utterance (Enfield, 2009; Ferrara and Johnston, 2014).

Relevant phenomena, such as constructed action in sign and demonstrations in speech, have both been analyzed as predicates, each providing action information that is not found anywhere else in the sentence. In the following examples, the first in sign (7) and the second in speech (8), the constructed action functions as a verb phrase:

(7) BOY (CA: THE BOY LOOKS INTO THE HOLE) Hodge and Johnston (2014)
(8) I got out of the car, and I just (*demonstration of turning around and bumping his head on an invisible telephone pole*) Clark and Gerrig (1990)

In the clause-like unit in (7), the actor is the boy, who is identified with the lexical sign BOY. However, there is no lexical verb describing the boy’s action of looking through the hole, other than the enactment in the constructed action. The signer uses his torso, head, and face to represent the boy, and enacts the looking process. In the clause-like unit in (8), the gestural demonstration also functions as a verb-like predicate, conveying action information that is not found in the speech. In our data, signers often use depiction as the only source of information about the size and shape of objects. These depictions thus appear to function as an adjectival predicate within the clause.

### Linguistic Constraints on Depictions

Handshapes in DCs have been analyzed as morphemes (Supalla, 1982, 1986) and we agree with this analysis. Indeed, both conventional and embellished DCs appear to be categorical (and conventional) in that the signers in our study always used the same handshape to refer to a particular type of object, for example, a G (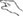) handshape to refer to small and discrete items (such as pills) or the Claw-5 (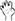) handshape to refer to round objects (such as rocks). Overall, there was very little variability in the handshape signers used to depict different shapes of objects, which suggests that handshape is conventional in these constructions (Frishberg, 1975; Kegl and Wilbur, 1976; Zwitserlood, 2003).

But handshape does not provide the full meaning of the object in embellished DCs (Zwitserlood, 2003). Signers added movement to the handshape to capture the referent’s shape. The movements they added were gradient (rather than categorical) and signers varied in the shapes they sculpted with their moving hands (Emmorey and Herzig, 2003; Liddell, 2003; Schembri et al., 2005). Nevertheless, there were still linguistic constraints on these embellished DCs. Signers could trace the contours of an object using either an index finger (which carves out a 2D representation) or another conventional handshape (which carves out a 3D representation). For the most part, signers chose the conventional handshape that captured a feature of the object. For example, signers used either a B (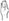) or Claw-5 (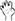) handshape to trace the contour of a vase, but did not use a less appropriate handshape such as a G (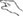) handshape. As another example, signers often used a C (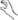) handshape to represent the stem of a mushroom and combined it with a Claw-5 (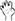) handshape to represent the cap; they rarely used other handshapes to represent this object. Handshape in an embellished DC is conventionally determined by the to-be-described object, following the same constraints that arise in conventional DCs. The key difference is that, in embellished (but not conventional) DCs, the handshape is modified with gradient movement or with another handshape to further specify the object’s shape.

Iconic mouth movements also provide shape information about objects, and may reflect an interaction between motoric and linguistic constraints. The mouth is not as free as the hands to convey shape through three-dimensional space. As a result, there are a limited number of mouth shapes available to signers that can function as conventional, adjectival mouth morphemes (Liddell, 1980; Sutton-Spence and Woll, 1999; Crasborn et al., 2008). For example, most signers puffed their cheeks when describing a vase. However, signers displayed variation in how they modified the *cheek puff* to capture the contour of the vase: one signer transitioned from a cheek puff to *ps*; another puffed multiple times to describe the same vase. The modifications overlaid on top of the mouth movements thus appear to be idiosyncratic and, in this sense, gestural.

Embellished DCs and modified iconic mouth movements can thus both be analyzed as categorical forms that are gradiently modified. These productions are comparable to analog speech in spoken language, where categorical forms are modified in a meaningful and iconic way (Shintel et al., 2006). For example, speech can be modified analogically by elongating the vowel “o” in the word *long:* “It was a *loooooong* time.” The conventionalized, categorical form “long” is modified gradiently to add emphasis to the meaning; it was not just a long time, but a *really* long time. However, there are constraints on how words can be modified analogically. For example, one cannot elongate other parts of the word and thus cannot say *lllllong* or *longngngng*; the vowel is a more likely candidate for modification than the consonant (Okrent, 2002). This constraint parallels the constraints that signers face when they use a particular handshape or mouth movement to construct a depiction.

### Depicting Constructions Share Properties With Spoken Ideophones

A special class of sensory words in spoken language—mimetics or ideophones found in African languages like Siwu or Japanese (Dingemanse and Akita, 2016)—may be a good analog to DCs in sign language. Like DCs, these spoken devices are iconic words that are borderline linguistic. They are flexible and amenable to gradient modification via reduplication or vowel lengthening. For example, the ideophone, *gat(-to)*, meaning a ‘rattling sound,’ can be reduplicated to *gagagagagagagat-to* to depict the reverberating sounds of debris falling (note that the morpheme within the ideophone, *gat*, can itself be iconic). Interestingly, ideophones frequently co-occur and are tightly integrated with iconic co-speech gestures, and often depict the same meaning as those gestures—they “perform the same role of depicting sensory imagery, albeit in different modalities and therefore also with different affordances for iconicity” (Dingemanse and Akita, 2016; see also Kita, 1997). For example, a speaker talks about an incoming wave using the ideophone, *zorot(-te)*, meaning ‘one after another in line,’ and reduplicates it, *zorozorot-te* while producing a time-aligned, reduplicated iconic gesture—he swings his arms from right to left twice.

Depicting constructions are similar to ideophones in two ways. First, categorical handshapes in signs are combined with non-discrete movement, just as conventional morphemes in ideophones are imagistically and analogically reduplicated, resulting in high expressivity. Second, DCs are tightly coupled with iconic mouth movements, just as ideophones are tightly coupled with co-speech gestures. If there is a spoken language that uses ideophones to describe the shape of objects, we predict that speakers of this language would use ideophones cohesively with iconic co-speech gesture.

### Conventions in the Practice of Depicting

We raise one last point with respect to depiction—although aspects of the form of depiction may not be entirely conventional, the practice of depicting may be conventional. The degree of conventionalization of a construction can be analyzed on two levels: conventionalization of the form itself (which we have discussed), and conventionalization of how the form is used (Okrent, 2002). Evidence from an emerging sign language shows that some linguistic devices can be conventionalized even before phonology emerges (Meir et al., 2010). In Al-Sayyid Bedouin Sign Language (ABSL), signers often use two signs to label a single object. For example, to identify a pen, signers often sign WRITE, followed by a conventional DC (‘SASS classifier’) referring to a thin object. Even though ABSL signers often choose different aspects of the pen to highlight in their conventional DCs, they are remarkably similar in how they order their two signs (DCs occupy the second position in 90% of instances).

In our data, depictions arise frequently, and we speculate, in a predictable way. For example, Enfield (2009) notes that there may be conventions with respect to when and how co-speech gestures are used (see also Lu and Goldin-Meadow, 2017). In fact, listeners can use the speaker’s eye gaze toward gesture space to predict when the speaker is going to produce a tracing gesture. Future work is needed to determine whether there is systematicity in when and how signers use embellished depictions, how embellished depictions relate to other constituents within the clause, and whether these depictions are foreshadowed by other cues.

### How Necessary Are DCs to Convey the Full Communicative Message?

Depicting constructions contribute significant meaning to linguistic utterance. Indeed, as in other depictive phenomena in spoken and signed languages (Clark and Gerrig, 1990; Liddell, 2003; Quinto-Pozos, 2007; Hodge and Johnston, 2014; Clark, 2016), the message would be incomplete without DCs. Signers may show a strong preference for depictive devices over lexical items in some communicative contexts simply because depictions can often provide more depth and accuracy in portraying a referent than lexical signs. In future work, our goal is to elicit judgments of signed utterances that contain either depiction or lexical items, and to assess how much information can be gleaned from these two types of utterances, how obligatory depictive devices are, and whether depictive devices provide richer meanings than descriptive devices.

We have demonstrated several ways in which depictive devices play an important role in conveying meaning in sign language. Importantly, this phenomenon is not unique to sign languages. Yet depiction tends to be relegated to the margins of language sciences and ignored in standard models of language (Liddell, 2003; Clark, 2016; Dingemanse and Akita, 2016). We have shown that signers, at times, will choose depiction over description as their primary communicative strategy, thus signaling the importance of depiction in discourse. The interesting question is whether depiction is just as important in spoken languages as it is in signed languages, a question that can only be answered by exploring depiction under comparable circumstances in speech and sign.

## Ethics Statement

All procedures for all studies reported were approved under University of Chicago IRB #15-1678. All participants provided written informed consent to participate in this study, and those identified in the images of the manuscript provided written informed consent for their publication.

## Author Contributions

JL and SG-M designed the research. JL performed the research and analyzed the data. JL and SG-M wrote the paper.

## Conflict of Interest Statement

The authors declare that the research was conducted in the absence of any commercial or financial relationships that could be construed as a potential conflict of interest. The reviewer RS and handling Editor declared their shared affiliation.

## Footnotes

1 Participants also saw 12 pairs of objects that were from the same category but of a different color (e.g., a white ornament vs. a green ornament) and thus could be identified by different color signs. The data from this ‘Descriptive (color)’ condition were not analyzed here.

2 For some figures, we have added information about iconic mouth movements using Anderson and Reilly’s (1998) notation system, denoted with underlines. For example, *puff* indicates that the cheeks were puffed out with air, and *ps* indicates that the cheeks were sucked in. There is also information about depicting constructions in brackets, which indicates the handshape being used and the type of movement involved in the production.
