# Supplementary material for: Creating Images With the Stroke of a Hand: Depiction of Size and Shape in Sign Language
Source: Front Psychol. 2018 Jul 31;9:1276. doi: 10.3389/fpsyg.2018.01276 (PMC6079389; doi:10.3389/fpsyg.2018.01276)
Supplement: Supplementary file 1 [file Table_1.docx]

*Table S1*

*Stimuli images used in the Description Elicitation Condition*

| Stimuli pair | Stimuli images | |
| --- | --- | --- |
| Clay vs. Ball | 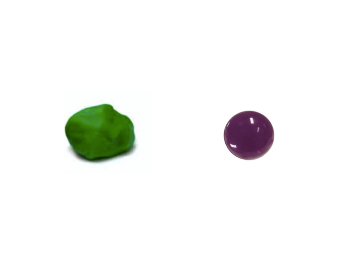 | |
| Cupcake vs. Dough | 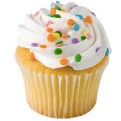 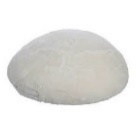 | |
| Broccoli vs. Lettuce | | 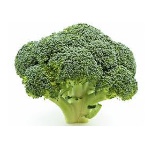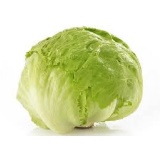 |
| French press vs. Olive oil | 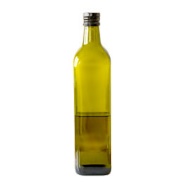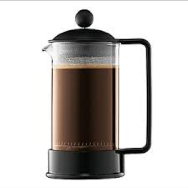 | |
| Pot vs. Bowl | 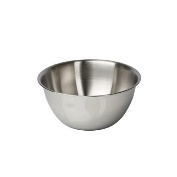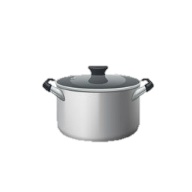 | |
| Glass vs. Can | 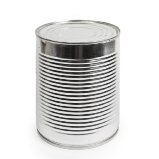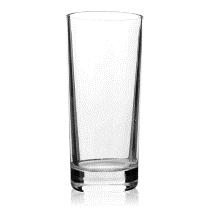 | |
| Pennies vs. Dimes | 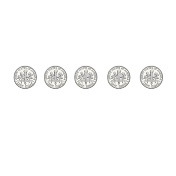 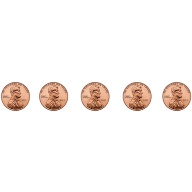 | |
| Ring vs. Button | 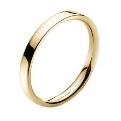 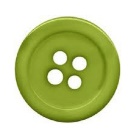 | |
| Jelly beans vs. Drops | 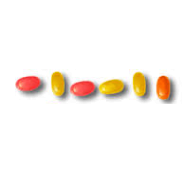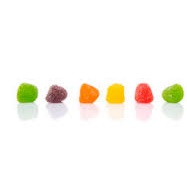 | |
| Screwdriver vs. Matchstick | 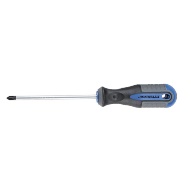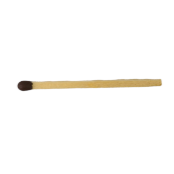 | |
| Bamboo vs. Straw | 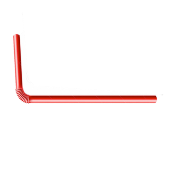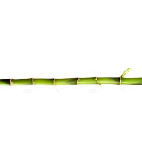 | |
| Ruler vs. Spoon | 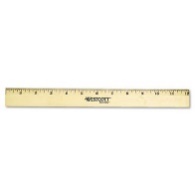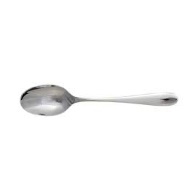 | |
