# Supplementary material for: Creating Images With the Stroke of a Hand: Depiction of Size and Shape in Sign Language
Source: Front Psychol. 2018 Jul 31;9:1276. doi: 10.3389/fpsyg.2018.01276 (PMC6079389; doi:10.3389/fpsyg.2018.01276)
Supplement: Supplementary file 2 [file Table_2.docx]

*Table S2*

*Stimuli images used in the Depiction Elicitation Condition*

| Stimuli pair | Stimuli images |
| --- | --- |
| Bacon 1 vs. Bacon 2 | 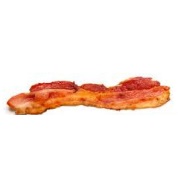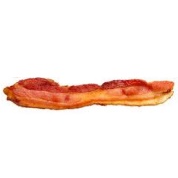 |
| Blue pills set 1 vs. Blue pills set 2 | 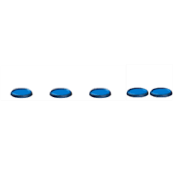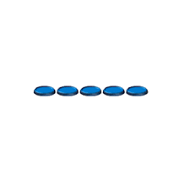 |
| Bottle 1 vs. Bottle 2 | 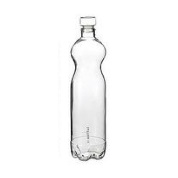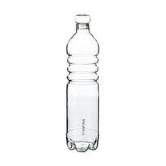 |
| Candle 1 vs. Candle 2 | 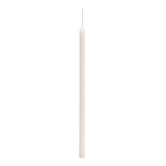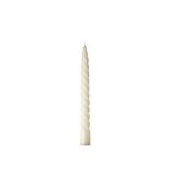 |
| Mushroom 1 vs. Mushroom 2 | 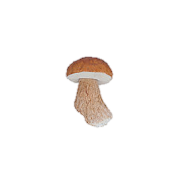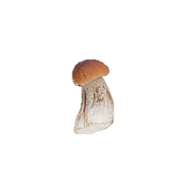 |
| Paperclips 1 vs. Paperclips 2 | 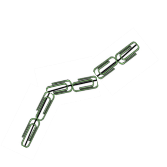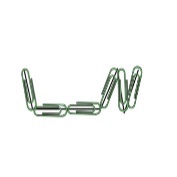 |
| Pitcher 1 vs. Pitcher 2 | 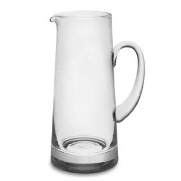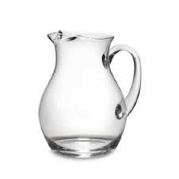 |
| Potato 1 vs. Potato 2 | 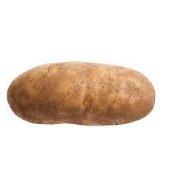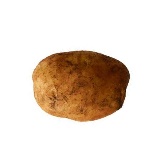 |
| Rock 1 vs. Rock 2 | 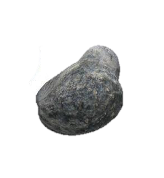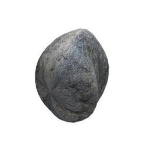 |
| Square set 1 vs. Square set 2 | 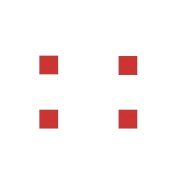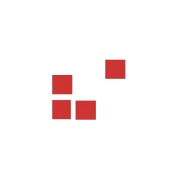 |
| Stick 1 vs. Stick 2 | 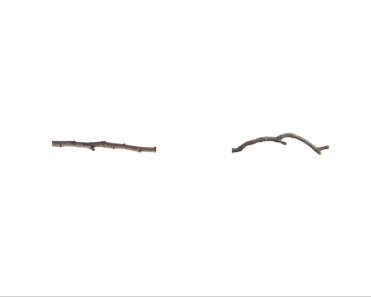 |
| Yellow vase 1 vs. Yellow vase 2 | 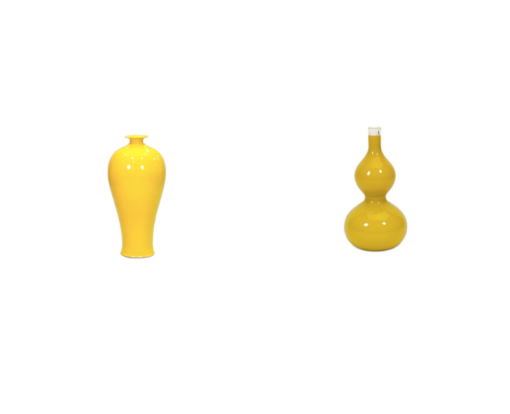 |
